# Quality Analysis and Correction of Sea Surface Temperature Data from China HY-1C Satellite in Southeast Asia Seas

**DOI:** 10.3390/s23187692

**Published:** 2023-09-06

**Authors:** Weifu Sun, Chalermrat Sangmanee, Yuanchi Jiang, Yi Ma, Jiang Li, Yujia Zhao

**Affiliations:** 1 Lab of Marine Physics and Remote Sensing, First Institute of Oceanography, Ministry of Natural Resources, Qingdao 266061, China; sunweifu@fio.org.cn; 2 Oceanography and Environment Division, Phuket Marine Biological Center, Phuket 830000, Thailand; 3 College of Oceanography and Space Informatics, China University of Petroleum (East China), Qingdao 266580, China

**Keywords:** SST, HY-1C, SST data evaluation, SST data correction

## Abstract

China’s marine satellite infrared radiometer SST remote sensing observations began relatively late. Thus, it is essential to evaluate and correct the SST observation data of the Ocean Color and Temperature Scanner (COCTS) onboard the China HY-1C satellite in the Southeast Asia seas. We conducted a quality assessment and correction work on the SST of the China COCTS/HY-1C in Southeast Asian seas based on multisource satellite SST data and temperature data measured by Argo buoys. The accuracy evaluation results of the COCTS SST indicated that the *bias*, *Std*, and *RMSE* of the daytime SST data for HY-1C were −0.73 °C, 1.38 °C, and 1.56 °C, respectively, while the *bias*, *Std*, and *RMSE* of the nighttime SST data were −0.95 °C, 1.57 °C, and 1.83 °C, respectively. The COCTS SST accuracy was significantly lower than that of other infrared radiometers. The effect of the COCTS SST zonal correction was most significant, with the *Std* and *RMSE* approaching 1 °C. After correction, the *RMSE* of the daytime SST and nighttime SST data decreased by 32.52% and 42.04%, respectively.

## 1. Introduction

Sea surface temperature (SST) is a fundamental physical parameter for studying sea–air interactions [1]. SST is a key indicator of changes in the Earth’s climate system [2,3,4,5]. SST has been widely used in various research, such as fisheries, ocean forecasting, meteorological forecasting, and marine environmental analysis [6,7]. The in situ measurements of SST are conducted through ships and buoys [8,9,10], and in situ SST measurements are usually accurate and unaffected by weather conditions. However, different platforms, measuring instruments, and data processing algorithms also bring uncertainties to the measured data [11], and the measured data lack continuity in time and space [12].

With the development of satellite remote sensing technology, acquiring the SST through spaceborne infrared and microwave radiometers has become the most important technical means for conducting large-scale SST observations and research [13]. The microwave radiometer signal has almost no attenuation after cloud penetration, so it can measure SST with a wider coverage [12], but the SST spatial resolution obtained is low. The infrared radiometer is easily blocked by clouds and affected by atmospheric aerosols [14], which makes it impossible to observe the sea surface under clouds [11], but it can achieve high-precision and high spatial resolution SST observations. With the great enrichment of spaceborne radiometer SST observation data, in recent years, SST data fusion, SST evaluation, and data analysis research continue to be studied.

High spatial resolution SSTs by infrared polar-orbiting satellites mostly include MODIS SSTs onboard Terra and Aqua; AVHRR SSTs onboard NOAA satellites; VIIRS SSTs onboard the Suomi NPP satellite [4]; ATSR SSTs flying onboard the ERS-1, ERS-2, and Envisat satellites; and SLSTR SSTs flown on the Sentinel-3 satellite (http://atsrsensors.org/aboutATSR.htm, accessed on 20 July 2023). China launched the HY-1C satellite on 7 September 2018, equipped with an Ocean Color and Temperature Scanner (COCTS) that can also provide SST remote sensing observation data [15]. As the third satellite in the Ocean One series, it has significantly enhanced its observation range compared to the HY-1A and HY-1B satellites [16]. The SST accuracy and spatial coverage obtained from different spaceborne infrared radiometers is different, so it is necessary to evaluate the accuracy of single-satellite infrared SST products.

The Southeast Asia seas are mostly composed of the eastern Indian Ocean, Andaman Sea, South China Sea, Malacca Strait, Sunda Strait, and other waterways [17]. Its special geographical location makes it one of the most important maritime trade routes in the world, and Southeast Asian countries have become the focus of China’s “Belt and Road” strategy [18]. Therefore, the practical application of China’s ocean satellite data in Southeast Asian countries is essential. However, there is currently limited research on the quality assessment and enhancement of China’s ocean satellite remote sensing SST data in Southeast Asian seas, which has affected the application of China’s ocean satellite data. We aimed to conduct a data quality analysis of the China HY-1C COCTS SST in Southeast Asia seas, understand the difference between the China HY-1C SST data and similar international satellite infrared radiometer SST data, carry out HY-1C satellite SST correction research based on international satellite SSTs, and provide correction methods for HY-1C satellite SST data to enhance the accuracy of the HY-1C satellite SST. This study is essential for improving the quality of China’s marine satellite SST products and enhancing the application level of SST products in Southeast Asian countries.

## 2. Data and Methods

### 2.1. Remote Sensing Data

The study area was the region between 20° S and 25° N latitude and 70° E and 150° E longitude. In this study, the SST data of AVHRR, MODIS-Aqua, MODIS-Terra, and the NPP VIIRS infrared radiometer in 2020 were selected for comparison with the China HY-1C COCTS SST, and the accuracy of infrared SST was compared in Southeast Asia seas. The data information is shown in Table 1. The HY-1C satellite was launched on 7 September 2018, which was the follow-up mission of the HY-1A and HY-1B satellites, with an orbital inclination of 98.5° [19]. The COCTS onboard the HY-1C satellite was a medium-resolution imaging scanner that detected global oceans and land twice a day, providing daily ocean color, land vegetation, and SST data for both the day and night [20]. Spectral specifications (spectral range, central wavelength, signal-to-noise ratio (SNR), noise-equivalent change in temperature (NEΔT), maximal radiance, and observation objective) of the COCTS/HY-1C are detailed in previous work [19]. The L2 SSTs of the COCTS onboard HY-1C are processed by the nonlinear SST algorithm (NLSST) using the brightness temperatures of the two COCTS thermal infrared bands at 10.8 and 12.0 µm [19].

The twice-daily 9 km SST data used in this study were sourced from the COCTS 3A standard product. The HY-1C SST products could be obtained for free from the Ocean Satellite Data Distribution System (OSDDS) of the National Satellite Ocean Application Service Center (NSOAS).

The AVHRR instruments onboard a series of satellites designed by NOAA [21] were often used to retrieve the SSTs. The SSTs from the AVHRR Pathfinder (PFV53) corresponded to a collection of global, twice-daily 4 km SST data, which were produced by the NOAA National Oceanographic Data Center and the University of Miami’s Rosenstiel School of Marine and Atmospheric Science. The MODIS instruments onboard the Terra and Aqua satellites are both currently in normal operation [22]. The MODIS 4 km/twice-daily SST data were obtained from the National Aeronautics and Space Administration (NASA). VIIRS (Visible Infrared Imaging Radiometer) is onboard the NPP satellite launched on 28 October 2011. It is an extension of and improvement in NASA’s AVHRR and MODIS series [23]. A better spatial resolution with a larger swath is available for VIIRS. NASA has provided a twice-daily available SST record of VIIRS with a resolution of 4 km. Table 1 introduces the infrared radiometers.

The quality level variable of the AVHRR SST ranged from 0 to 7. For the quality variable, higher was better, and therefore, 0 was worst and 7 was best. In this study, data with a quality level of 7 and a valid range of −1.8 to 45 °C were used. The quality level of the MODIS and VIIRS SST could theoretically range from 0 (best) to 4 (worst). The valid range of the data was −2 to 45 °C. We retained the data with the quality level marked as 0 in the valid SST range.

### 2.2. Argo Buoy Observation Data

**Figure 1:**
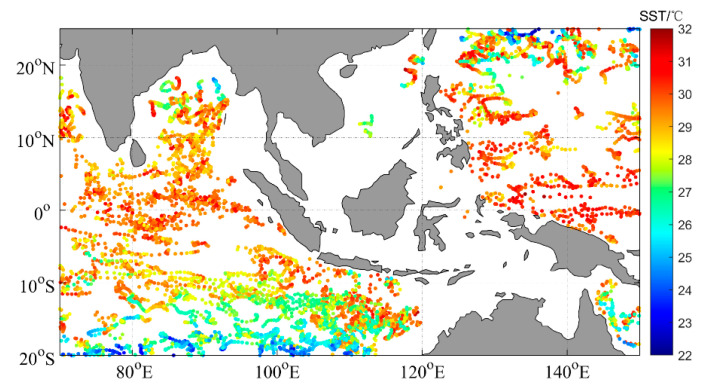
The distribution of Argo buoy SSTs in 2020.

The international Argo (Array for Real-time Geostrophic Oceanography) program aims to establish a global ocean observation network composed of drifting buoys with a spacing of approximately 3° × 3°. Each Argo float collects temperature and salinity profiles (approximately 1000 data points) of seawater in the depth range of 0–2000 m once every ten days. In this study, the accuracy of infrared radiometer SST data was validated using Southeast Asian seas Argo temperature data for the entire year of 2020. Due to the scarcity of buoy observation data with depths between 0 and 1 m, the temperature data from buoys at depths between 0 and 10 m were selected to ensure that the data analysis was statistically significant [4,13,24]. A total of 7171 valid float data points within the study area (0–10 m water depth) were selected from global Argo floats, distributed as shown in Figure 1. The SST values ranged from 22.3 °C to 32.3 °C, and the floats were mostly concentrated in the Indian Ocean and the western Pacific Ocean east of 120° E. There was relatively little Argo data coverage in the South China Sea, as only a few floats could enter this area due to the influence of the current field in the Luzon Strait.

### 2.3. Quality Evaluation Criteria for SST Data

The accuracy of the spaceborne infrared radiometer SST was evaluated by spatiotemporal matching with Argo float temperature data in 2020. The evaluation was based on several metrics, including the mean deviation (*bias*), standard deviation (*Std*), root-mean-square error (*RMSE*), and correlation coefficient (*R*), which were calculated between the satellite SST data from AVHRR, MODIS-Aqua, MODIS-Terra, and HY-1C COCTS and the Argo float data. The expressions of the statistical parameters are as follows:

(1) $$Bias=\frac{1}{N}\sum_{i=1}^{N}\left(A_{i}-S_{i}\right)$$

(2) $$Std=\sqrt{\frac{\sum_{i=1}^{N}{\left[(A_{i}-S_{i})-(\bar{A-S})\right]}^{2}}{N}}$$

(3) $$\mathrm{RMSE}=\sqrt{\frac{1}{N}\sum_{i=1}^{N}{\left(A_{i}-S_{i}\right)}^{2}}$$

(4) $$R=\frac{\sum_{i=1}^{N}\left[(A_{i}-\bar{A})-(S_{i}-\bar{S})\right]}{\sqrt{\sum_{i=1}^{N}{\left(A_{i}-\bar{A}\right)}^{2}\sum_{i=1}^{N}{\left(S_{i}-\bar{S}\right)}^{2}}}$$

where *N* represents the total number of matching data of SSTs from remote sensing observations and SSTs from Argo observations, *A* represents remote sensing observations, and B represents SST values from Argo observations.

The temporal and spatial matching windows for AVHRR, MODIS-Aqua, MODIS-Terra, and the HY-1C COCTS were set at 12 h and 0.09°, respectively. According to the 3-sigma principle, we considered the matched data with deviations beyond *bias* ± 3*Std* as outliers and removed them.

## 3. Analysis of the HY-1C Satellite SST Data Quality

**Figure 2:**
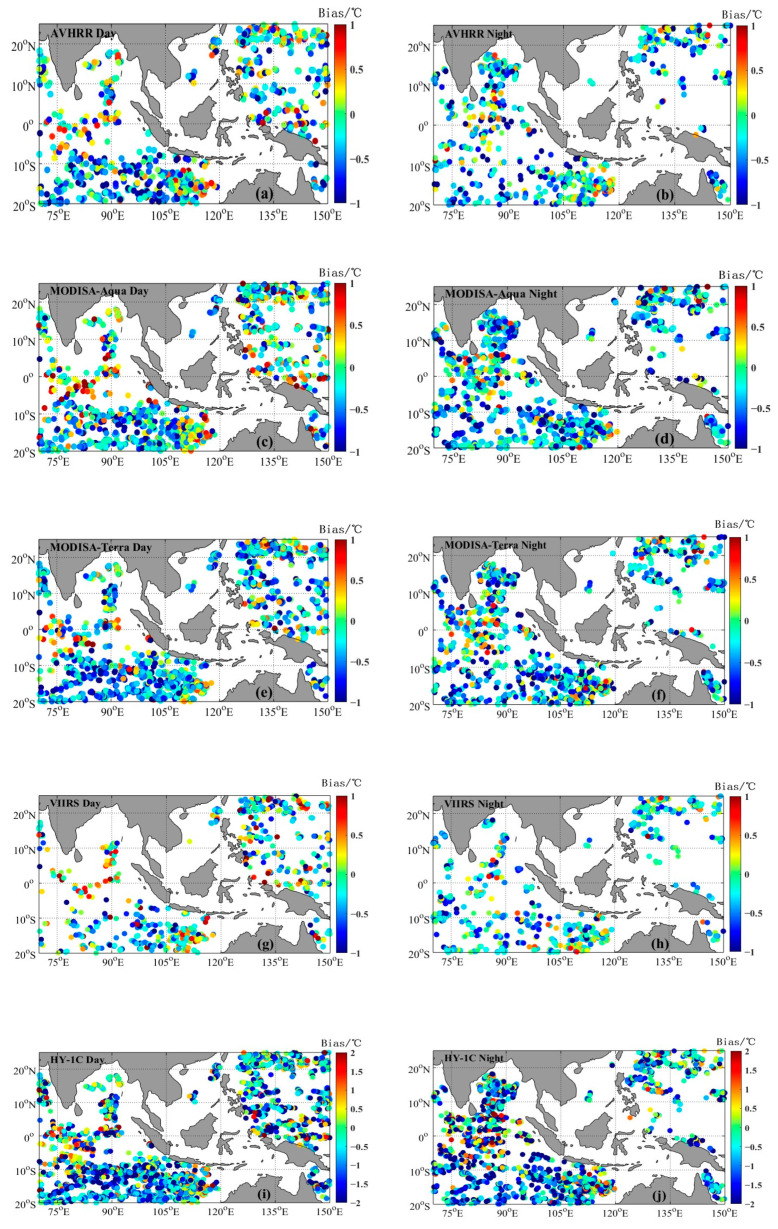
Distribution of matched points between infrared radiometer SST and Argo observations in Southeast Asia Seas.

The matched point positions between the infrared SST and buoy-measured data are shown in Figure 2. As shown in Figure 2, the matched points were mainly distributed in the eastern Indian Ocean and western Pacific Ocean, while there were fewer matching points in the surrounding waters of the South China Sea, which was related to the distributions of the Argo buoys. There was an overall negative deviation between the satellite SST and Argo data. The negative deviation values with significant deviation were mostly concentrated between latitudes 20° S and 10° S, while the positive deviation values (red dots) were mainly in the Indian Ocean waters between latitudes 10° S and 10° N.

The large deviation was caused by the difference between the satellite SST and underwater temperature measured by Argo buoys. The underwater temperature was relatively stable, and the SST was related to the solar elevation angle. The equatorial region was affected by solar radiation during the daytime, resulting in an SST exceeding the buoy temperature, so there was a significant positive deviation in the equatorial region during the daytime. The nighttime coolness in the slight Highlands area resulted in the SST being less than the buoy temperature, so there was a significant negative deviation [25].

**Figure 3:**
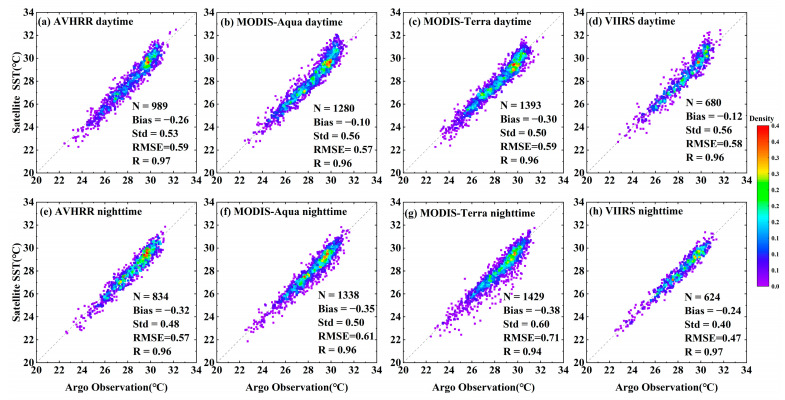
Scatter plot of AVHRR, MODIS, and VIIRS SST and Argo observation data.

**Figure 4:**
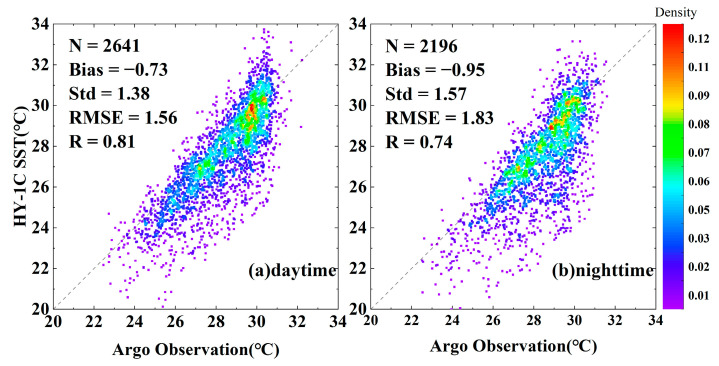
Scatter plot of HY-1C SST and Argo observation data.

The error statistics between the satellite SST of five infrared radiometers and Argo observations are shown in Table 2. The *bias* of the five infrared radiometers was negative, and the absolute value of the *bias* of each radiometer at night was greater than that in the daytime. The *bias* of the MODIS-Aqua and VIIRS daytime SST data was the smallest, with values of −0.1 °C and −0.12 °C, respectively. Except for the HY-1C COCTS, MODIS-Terra had the highest nighttime *Std* at 0.6 °C, while the *RMSE* was also relatively large, reaching 0.71 °C. The scatter plots of the AVHRR, MODIS-Aqua, MODIS-Terra, and VIIRS remote sensing SST and Argo-measured data are shown in Figure 3. From Figure 3, it can be observed that the *bias*, *Std*, and *RMSE* of the MODIS-Terra nighttime SST were greater than those of the daytime data, while the *Std* and *RMSE* of the VIIRS nighttime SST were smaller than those of the daytime data. The four satellite SSTs had good correlation with the Argo data in the Southeast Asia seas, with the VIIRS SST data having the best quality. During the daytime, the *bias*, *Std*, and *RMSE* of HY-1C were −0.73 °C, 1.38 °C, and 1.56 °C, respectively. The data quality during the nighttime was lower than that during the daytime, while the *bias*, *Std*, and *RMSE* were −0.95 °C, 1.57 °C, and 1.83 °C, respectively. The scatter plot of the HY-1C SST and Argo-measured data is shown in Figure 4. Overall, the deviation between the HY-1C SST and Argo-measured temperature was significantly larger compared to other satellite SSTs.

**Figure 5:**
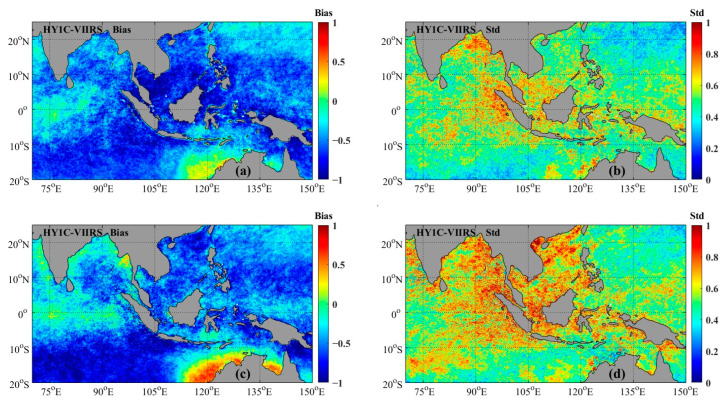
Spatial distribution of SST deviation between HY-1C SST and VIIRS SST ((**a**,**b**): daytime; (**c**,**d**): nighttime).

As mentioned above, the VIIRS SST has the best data quality. Therefore, we will use the VIIRS SST to calibrate the HY-1C SST. Before data correction, we conducted cross validation between the HY-1C SST data and VIIRS SST data. The VIIRS SST data were interpolated into a grid consistent with the HY-1C SST data using bilinear interpolation. Monthly average SST data were calculated based on the unified grid-based HY-1C SST and VIIRS SST data. The distribution of the *bias* and *Std* of the monthly average SST for HY-1C and VIIRS in 2020 is shown in Figure 5. As shown in Figure 5, the difference between HY-1C and VIIRS was mostly negative in most areas. During the daytime, the absolute value of the *bias* was relatively large near the South China Sea (with a latitude range of 10° S~10° N), and at nighttime, the absolute value of the *bias* was the highest in the Indian Ocean at a latitude of 20° S~10° S. Figure 5b,d show the larger *Std* near the equator, the Bay of Bengal, and the South China Sea. Considering the distribution characteristics of the HY-1C SST deviation, subdividing the region and developing different correction relationships for different regions could enhance the accuracy of the HY-1C SST.

To investigate the seasonal correlation and the differences between the HY-1C SST and VIIRS SST, the *bias* and *Std* of the monthly average SST data in Southeast Asian waters were calculated, and the statistical results are shown in Table 3. Table 3 shows that the *bias* between the HY-1C SST and VIIRS SST was negative. The daytime and nighttime data of the HY-1C SST and VIIRS SST fluctuated between −0.69 °C to −0.37 °C and −0.80 °C to −0.16 °C, respectively, indicating that the COCTS/HY-1C SST was lower than the VIIRS SST and had seasonal characteristics. The cloud detection processing method for the COCTS is not effective enough for some conditions, such as light cloud covering, so some gridded SSTs of the COCTS become lower. A more effective cloud detection method for the COCTS needs to be developed in the future [26].

The *Std* values of the HY-1C daytime and nighttime SST were between 0.47~0.69 °C and 0.52~0.76 °C, respectively. The *Std* values in the second half of the year were larger than those in the first half of the year, and the *Std* values at night were higher than those during the daytime. In December, the *bias* and *Std* values at night deviated most significantly, reaching −0.8 °C and 0.76 °C, respectively. The deviation of the HY-1C SST and VIIRS SST had a certain correlation with the daytime, nighttime, and season. Therefore, correction studies could be conducted on the HY-1C daytime and nighttime SST by month.

**Figure 6:**
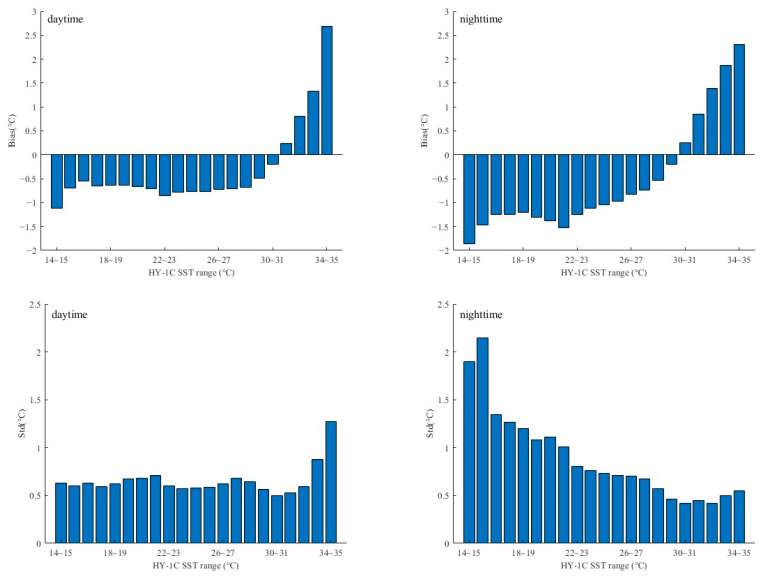
Bias and Std between HY-1C and VIIRS SST during daytime and nighttime in different SST ranges.

In this study, we segmented the HY-1C SST data at intervals of 1 °C and conducted a matching analysis using the monthly average SST data of the HY-1C COCTS and VIIRS. The *bias*, *Std*, and number of matching points for different SST segments were calculated. The statistical results are shown in Table 4 and Figure 6. It can be seen that the SSTs below 16 °C and above 34 °C have limited matching data and larger deviations. After removing the outliers with large deviations and few matches, for the HY-1C SST, 16~34 °C was selected as the effective SST data, and the range was divided into 18 segments at 1 °C intervals to conduct calibration research on the HY-1C SST data. Table 4 shows that the *bias* of the HY-1C SST was negative in the range of 16 °C to 30 °C and positive in the range of 31 °C to 34 °C, indicating that the HY-1C SST was lower than the VIIRS SST value when it was below 30 °C and higher in high-temperature conditions. Therefore, correcting the SST for different temperature ranges can enhance the quality of the HY-1C data.

## 4. Correction of HY-1C Satellite SST Data

As mentioned above, the accuracy of several satellite infrared radiometer SST products was evaluated using Argo observation data, and it was found that there was still a certain gap in data accuracy between China’s HY-1C satellite and the international mainstream satellite SST. Among them, the VIIRS SST had the highest accuracy, so we chose VIIRS for the data correction of the HY-1C satellite SST.

### 4.1. SST Correction Based on Monthly Bias

Based on the error analysis of the HY-1C SST in different months in the previous text, it was found that the HY-1C SST had a significant seasonal variation trend. Through an evaluation study based on Argo data, it was discovered that the SST of HY-1C still had a certain distance from the international satellite SST. We developed a monthly correction algorithm for the HY-1C SST based on the regularity of the deviation between the HY-1C SST and AMSR2 SST for different months. The correction values for the HY-1C monthly mean SST were calculated using Equation (5).

Through evaluation research based on Argo observations, it was found that there was still a certain shortcoming between the HY-1C SST and other infrared SSTs. This study developed a monthly correction algorithm for the HY-1C SST based on the deviation characteristics between the HY-1C SST and VIIRS SST in different months. Equation (5) was used to calculate the correction value for the monthly average SST of HY-1C.

(5) $$B_{HC}(i)=S_{V}\left(i\right)-S_{HC}\left(i\right)$$

In Equation (5), *i* represents the grid position; *B_HC_* represents the corrected SST value of HY-1C; *S_V_* represents the monthly average SST value of VIIRS; and *S_HC_* represents the monthly average SST value of the HY-1C satellite.

For the comparative analysis, we also calculated the annual average deviation of the HY-1C SST and VIIRS SST, which was used as the system deviation of the HY-1C satellite and applied to the daily SST throughout the year. For data corrected for monthly and annual mean deviations, the Argo buoy data were used for the accuracy evaluation. The SST error statistics before and after the HY-1C correction are shown in Table 5.

Table 5 shows that the *bias* of the SST data after correction significantly decreased, and the *RMSE* was also partially reduced. Through the annual average correction method, the *RMSE* of the SST during the daytime decreased by 11.46%, and the *RMSE* at night decreased by 14.01%. Through the monthly average correction method, the *RMSE* of the SST decreased by 17.4% during the daytime and 19.38% at night, indicating that HY-1C could achieve enhanced effects through the monthly average correction method.

### 4.2. SST Correction Carried out across Different Regions

Due to the seasonal characteristics of the deviation between the HY-1C SST and VIIRS SST, we used the least squares linear regression method to establish a monthly empirical model between the HY-1C SST and VIIRS SST and then calculated the corrected HY-1C SST. The quality of the data before and after correction was evaluated using Argo data.

The monthly regression model for the HY-1C SST was obtained using the linear regression model, as shown in Equations (6) and (7).

(6) $$S_{HC}\prime =\alpha \times S_{HC}+\beta$$

In Equation (6), S_HC_ represents the observed SST values from the HY-1C satellite; $S_{HC}\prime$ represents the calculated SST value based on the correction model; β is the intercept of the regression model; and α is the slope of the regression model. The coefficients α and β are obtained based on the least squares method.

(7) $$\begin{array}{l} \beta =\frac{\sum S_{V}}{n}-\alpha \frac{\sum S_{HC}}{n}=\bar{S_{V}}-\alpha \bar{S_{HC}}\\ \alpha =\frac{n\sum S_{HC}S_{V}-\sum S_{HC}\sum S_{V}}{n\sum S_{HC}{}^{2}-{\left(\sum S_{HC}\right)}^{2}} \end{array}$$

In Equation (7), S_HC_ represents the observed SST values from HY-1C, *S_V_* is the corresponding SST value from VIIRS for the same grid point, n is the number of matching points, and $\bar{S_{V}}$ and $\bar{S_{HC}}$ are the mean values of *S_V_* and *S_HC_*, respectively.

Equations (6) and (7) were used to calculate the monthly correction model parameters of the HY-1C SST, and the results are shown in Table 6. The model was applied to calibrate daily HY-1C SST data. Argo data were used to evaluate the corrected SST data. An error analysis was conducted on the matching results to evaluate the quality of the corrected data. The statistical results of correcting SST errors are shown in Table 7.

The results showed that the linear regression method significantly improved the accuracy of HY-1C, with the *RMSE* decreasing to 1.2266 °C and 1.3134 °C during the daytime and nighttime, respectively. Compared to before correction, the error decreased by 21.35% and 28.31%, respectively.

**Figure 7:**
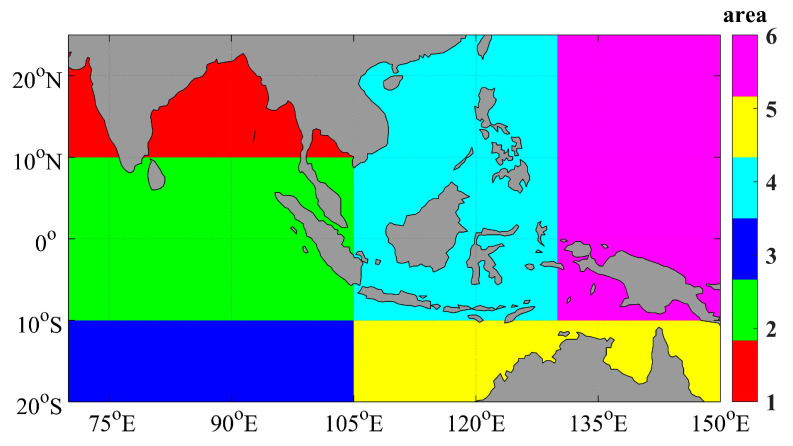
Regional Division of Southeast Asia Seas.

From Figure 5, it can be seen that the larger positive deviations are in the northern waters of Australia, and the smaller positive deviations are in the Bay of Bengal, the waters of western Western India, and the waters of southern Sri Lanka. The significant negative deviations are found in the southwest waters of the study area and the South China Sea. The smaller negative deviations are located in the northeast sea area of the study area. Based on the spatial distribution of deviations within the study area, the scope of the Southeast Asian seas was divided. The refined regional distribution corrected for HY-1C in this article is shown in Figure 7.

In this study, we used a linear regression method, accounting for subregional differences, to construct separate empirical relationships for each subregion to accurately correct the HY-1C SST. We used a consistent spatiotemporal window and evaluated the accuracy by matching the corrected SST with Argo observations. The statistical results of the error analysis are shown in Table 7. The calibration model parameters for region 1 are shown in Table 8. 

After the HY-1C SST regional correction, the daytime and nighttime biases were −0.1352 °C and −0.3473 °C, respectively, and the RMSEs were 1.0524 °C and 1.0618 °C, respectively. Compared with the precorrection accuracy, the effect was significant, with the *RMSE* reduced by 32.52% and 42.04%, respectively. Compared with nonregional correction, the *RMSE* of the night SST data decreased by 0.2516 °C. Overall, the subregional correction method had the greatest performance, with the most significant data correction effect at night. After regional correction, the issue of significantly lower nighttime SST data quality than daytime data quality was improved, resulting in a more balanced daytime and nighttime data quality for HY-1C.

### 4.3. SST Correction within Each Range of 1 °C

Table 4 shows that the deviation varied between different SST ranges. In this section, we divided the HY-1C SST range into 18 segments (SST range was 16~34 °C, with a 1 °C interval between each segment) and performed segmented calibration of the HY-1C SST data. The SST range here was the HY-1C SST range (i.e., the satellite values that need to be corrected). Due to the correlation between months and deviations, different segmented SST linear correction models were obtained using the SST from different months (with an interval of 1 °C), and the models were applied to the daily SST data of HY-1C to obtain the HY-1C correction SST dataset. The correction results were evaluated using Argo observation data, and the error statistics are shown in Table 9. The statistical results showed that the *RMSE* of the HY-1C daytime and nighttime SST was 1.2040 °C and 1.3331 °C, respectively, with an improvement of 22.8% in daytime accuracy and 27.23% in nighttime accuracy.

### 4.4. Comparison of SST Correction Results

**Figure 8:**
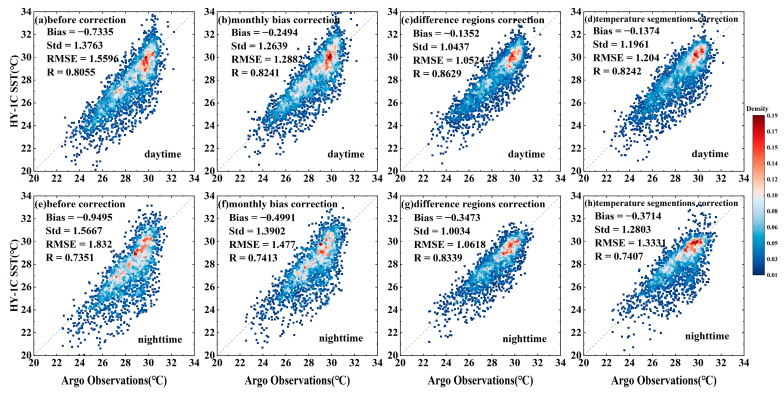
Comparison of HY-1C SST with Argo data before and after correction.

Based on the VIIRS SST data, the HY-1C SST was calibrated using methods of monthly average bias correction, different region correction, and temperature segmentation correction. The scatter plot of the comparison between the HY-1C SST (before and after correction) and Argo-measured SST is shown in Figure 8. All three correction methods improved the HY-1C SST data, but there were still significant errors. The effect of regional correction was the best, with the *Std* and *RMSE* close to 1 °C after correction, and the *RMSE* decreased by approximately 0.8 °C at night compared to before correction.

**Figure 9:**
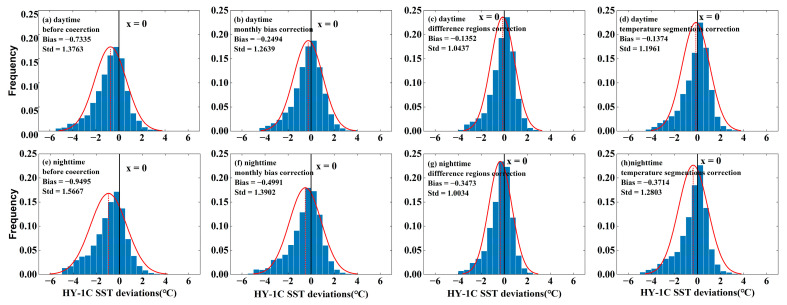
Distribution of daytime and nighttime SST difference between HY-1C SST and Argo data before and after correction.

The statistical histogram of the deviation between the HY-1C SST and Argo-matching data before and after correction is shown in Figure 9. From Figure 9, it is observed that before the SST data correction, 82.44% of the daytime SST differences were within ±2 °C, 56.58% of the SST differences were within ±1 °C, and the proportion of data with SST deviations within the ±0.5 °C range was 32.57%. The nighttime SST data were worse than those during the daytime, with 78.9% of the SST differences within ±2 °C before correction, 53.23% within ±1 °C, and 30.86% within the ±0.5 °C deviation range. Through regional correction, the deviation distribution was more concentrated than before, with 92.79% of the daytime data deviation within ±2 °C, 72.14% of the daytime data deviation within ±1 °C, and the SST deviation within the ±0.5 °C range reaching 42.95%. For the nighttime SST, 92.3% of the data after regional correction had a deviation within ±2 °C, 72.59% of the data had a deviation within ±1 °C, and 45.74% of the data were corrected to within ±0.5 °C. The results indicated that the HY-1C SST had been effectively improved through regional correction.

## 5. Conclusions

Using Argo-measured temperature data, the satellite-borne infrared radiometer SST data of AVHRR, MODIS-Aqua, MODIS-Terra, VIIRS, and the HY-1C COCTS were evaluated, and the differences between China’s HY-1C SST and the international similar satellite SST were analyzed. The HY-1C SST was calibrated using three calibration methods (monthly average bias correction, different region correction, and temperature segmentation correction) using the VIIRS SST. The major conclusions are as follows:

The accuracy evaluation results showed that the correlation coefficient between AVHRR, MODIS-Aqua, MODIS-Terra, the VIIRS SST, and the Argo observation data was better than 0.93, and the *RMSE* was better than 0.71. Among them, VIIRS had the highest accuracy, with a nighttime SST *RMSE* of 0.4 °C. During the day, the *bias*, *Std*, and *RMSE* of HY-1C SST were −0.73 °C, 1.38 °C, and 1.56 °C, respectively. At night, the *bias*, *Std*, and *RMSE* of HY-1C were −0.95 °C, 1.57 °C, and 1.83 °C, respectively, indicating significant differences between the SST of AVHRR, MODIS-Aqua, MODIS-Terra, and VIIRS.

Based on the VIIRS SST, three calibration methods were used to calibrate the HY-1C SST. The results showed that the accuracy improvement in the HY-1C SST after regional correction was the most significant compared to before correction, with the *Std* and *RMSE* close to 1 °C. The *RMSE* of the SST at night decreased by approximately 0.8 °C compared to before correction. The *RMSE* of the SST during the daytime and nighttime decreased by 32.52% and 42.04%, respectively.

## Figures and Tables

**Table 1:** Infrared radiometer SST products used in this study.

Satellite Sensor	Orbital Inclination	Channels Used for SST Retrieval	Spatiotemporal Resolution
AVHRR	98.7°	10.8 and 11.4 µm	4 km × 4 km
MODIS-Aqua	98.2°	11.0 and 12.0 µm	4 km × 4 km
MODIS-Terra	98.2°	11.0 and 12.0 µm	4 km × 4 km
NPP VIIRS	98.7°	10.8 and 12.0 µm	4 km × 4 km
HY-1C COCTS	98.5°	10.8 and 12.0 µm	9 km × 9 km

**Table 2:** Statistical analysis of errors in satellite infrared radiometer SST.

Sensors	Daytime					Nighttime				
	Num	Bias (°C)	Std (°C)	RMSE (°C)	R	Num	Bias (°C)	Std (°C)	RMSE (°C)	R
AVHRR	989	−0.26	0.53	0.59	0.9650	834	−0.32	0.48	0.57	0.9623
Aqua	1280	−0.10	0.56	0.57	0.9592	1338	−0.35	0.50	0.61	0.9575
Terra	1393	−0.30	0.50	0.59	0.9647	1429	−0.38	0.60	0.71	0.9391
VIIRS	680	−0.12	0.56	0.58	0.9575	624	−0.24	0.40	0.47	0.9744
HY-1C	2641	−0.73	1.38	1.56	0.8055	2196	−0.95	1.57	1.83	0.7351

**Table 3:** Statistics on monthly average SST difference between HY-1C and VIIRS.

Month	Daytime		Nighttime	
	Bias (°C)	Std (°C)	Bias (°C)	Std (°C)
January	−0.51	0.47	−0.52	0.52
February	−0.60	0.54	−0.58	0.60
March	−0.47	0.54	−0.23	0.60
April	−0.37	0.51	−0.16	0.57
May	−0.54	0.59	−0.47	0.67
June	−0.45	0.54	−0.46	0.62
July	−0.56	0.60	−0.62	0.67
August	−0.53	0.57	−0.47	0.69
September	−0.62	0.64	−0.59	0.69
October	−0.59	0.67	−0.62	0.75
November	−0.49	0.63	−0.48	0.69
December	−0.69	0.69	−0.80	0.76

**Table 4:** Statistics of the deviation between HY-1C SST and VIIRS SST for different SST ranges.

HY-1C SST Range (°C)	Daytime			Nighttime		
	Bias (°C)	Std (°C)	Num	Bias (°C)	Std (°C)	Num
14~15	−1.1210	0.6306	9	−1.8572	1.8957	149
15~16	−0.7007	0.6044	127	−1.4614	2.1473	371
16~17	−0.5503	0.6320	445	−1.2469	1.3451	737
17~18	−0.6483	0.5929	725	−1.2492	1.2678	1135
18~19	−0.6419	0.6238	1150	−1.2097	1.1955	1816
19~20	−0.6365	0.6728	1808	−1.3089	1.0812	2751
20~21	−0.6637	0.6797	2647	−1.3877	1.1086	4347
21~22	−0.7074	0.7086	4871	−1.5235	1.0099	10,864
22~23	−0.8607	0.6006	22,901	−1.2480	0.8037	46,405
23~24	−0.7869	0.5736	78,479	−1.1212	0.7593	103,728
24~25	−0.7635	0.5759	149,014	−1.0460	0.7289	183,615
25~26	−0.7661	0.5889	274,993	−0.9746	0.7081	316,240
26~27	−0.7285	0.6204	457,250	−0.8282	0.7036	521,043
27~28	−0.7074	0.6790	614,255	−0.7404	0.6736	713,743
28~29	−0.6794	0.6435	971,548	−0.5334	0.5705	1,133,574
29~30	−0.4871	0.5614	1,274,298	−0.2013	0.4659	1,123,441
30~31	−0.1957	0.4983	673,177	0.2514	0.4217	378,648
31~32	0.2432	0.5265	104,214	0.8516	0.4452	47,236
32~33	0.7999	0.5955	8667	1.3840	0.4225	6445
33~34	1.3271	0.8789	766	1.8719	0.5026	456
34~35	2.6796	1.2683	84	2.3029	0.5471	27

**Table 5:** Statistical results of SST error of HY-1C monthly average correction.

	Orbit	Bias (°C)	Std (°C)	RMSE (°C)	RMSE Reduction
Before calibration	Daytime	−0.7335	1.3763	1.5596	-
	Nighttime	−0.9495	1.5667	1.8320	-
Annual average calibration	Daytime	−0.3128	1.3450	1.3809	11.46%
	Nighttime	−0.4780	1.5012	1.5754	14.01%
Monthly average calibration	Daytime	−0.2494	1.2639	1.2882	17.4%
	Nighttime	−0.4991	1.3902	1.4770	19.38%

**Table 6:** Monthly correction model parameters for HY-1C SST.

Month	Daytime		Nighttime	
	Slope	Intercept	Slope	Intercept
January	0.9515	1.8788	0.8421	4.8759
February	0.9415	2.2417	0.8596	4.4321
March	0.9248	2.6435	0.8222	5.2680
April	0.8867	3.6729	0.8030	5.8395
May	0.8953	3.5721	0.7460	7.7420
June	0.9100	3.0507	0.8081	5.9227
July	0.9145	2.9962	0.8312	5.3515
August	0.8963	3.4813	0.8134	5.6887
September	0.9042	3.3464	0.8226	5.5399
October	0.8496	4.8609	0.7592	7.33
November	0.8218	5.5914	0.7234	8.2583
December	0.8962	3.6265	0.7705	7.1341

**Table 7:** Statistical results of the HY-1C subarea correction SST error based on Argo.

	Orbit	Bias (°C)	Std (°C)	RMSE (°C)	RMSE Reduction
Before calibration	Daytime	−0.7335	1.3763	1.5596	-
	Nighttime	−0.9495	1.5667	1.8320	-
Linear regression	Daytime	−0.0832	1.2238	1.2266	21.35%
	Nighttime	−0.3214	1.2735	1.3134	28.31%
Regional calibration	Daytime	−0.1352	1.0437	1.0524	32.52%
	Nighttime	−0.3473	1.0034	1.0618	42.04%

**Table 8:** Monthly correction model parameters for HY-1C SST in region 1.

Month	Daytime		Nighttime	
	Slope	Intercept	Slope	Intercept
January	0.9520	1.6179	0.9542	1.5716
February	0.9267	2.3031	0.9685	1.1034
March	1.0320	−0.6949	0.9874	0.1839
April	0.9248	2.3884	0.7983	5.6963
May	0.5259	14.7536	0.4081	17.9935
June	0.2199	23.4253	0.1995	23.8084
July	0.2882	21.3061	0.2055	23.4579
August	0.3867	18.1932	0.3265	19.5316
September	0.4476	16.6726	0.3295	19.6555
October	0.4389	16.5605	0.3195	19.6818
November	0.3918	17.8971	0.2278	22.3584
December	0.8543	4.4553	0.7635	11.0505

**Table 9:** Statistical results of segmented correction errors for HY-1C SST.

	Orbit	Bias (°C)	Std (°C)	RMSE (°C)	RMSE Reduction
Before calibration	Daytime	−0.7335	1.3763	1.5596	-
	Nighttime	−0.9495	1.5667	1.8320	-
Segmented correction	Daytime	−0.1374	1.1961	1.2040	22.8%
	Nighttime	−0.3714	1.2803	1.3331	27.23%

## Data Availability

The SST data of MODIS and VIIRS can be obtained on the official oceancolor website of NASA (https://oceancolor.gsfc.nasa.gov/). The AVHRR SST can be obtained on the website of https://www.ncei.noaa.gov/data/oceans/pathfinder/Version5.3/L3C/. The HY-1C SST data can be obtained by making official requests to NSOAS. The results data can be found at this link (https://pan.baidu.com/s/16OgrOQaSWn1M6RT_eVnEJA; the extracted code is ‘tjrh’).
